# CST1 promoted gastric cancer development by activating the AKT pathway

**DOI:** 10.1016/j.clinsp.2024.100561

**Published:** 2024-12-24

**Authors:** Lei Zhang, Dongmei Wang, Liqun Zhang, Lini Zhu

**Affiliations:** a Department of Gastroenterology, The First Affiliated Hospital of Soochow University, Soochow, China; b Department of Gastroenterology, Punnan Branch of Renji Hospital, Shanghai Jiaotong University School of Medicine, Shanghai, China

**Keywords:** Gastric cancer, CST1, AKT

## Abstract

**Background:**

Gastric Cancer (GC) was the third highest mortality rate among malignant tumors. Currently, no specific treatment is utilized to prevent the progression of GC. The detailed mechanism of GC was still elusive and this study aimed to clarify the mechanism of GC occurrence and development.

**Method:**

This study was performed to clarify the molecular mechanisms of CST1 promoting GC development through activating AKT. The normal gastric tissue cells and GC cell was obtained, followed by transfection with oe-CST1 or sh-CST1, and their apoptosis and viability were evaluated. Finally, Western blot, Flow cytometry assay, Transwell assay, and Scratch assay were used to elucidate the molecular mechanisms of CST1 promoting GC development through activating the AKT pathway.

**Results:**

Research outcomes show a significant elevation in CST1 and AKT protein as well as mRNA quantities in both the model and CST1-activator cohorts in relation to the control. Conversely, these proteins and mRNA concentrations were notably decreased in the presence of the CST1 inhibitor when compared to the model group, a difference that was statistically significant as evidenced by the *p*-value.

**Conclusion:**

CST1 can promote the gastric cancer process by targeting the AKT pathway.

## Highlights

• Comparison of the invasive capabilities.
• Comparison of the metastatic capabilities.
• Effect of CST1 and AKT mRNA in gastric cancer.
• Effect of CST1 and AKT protein in gastric cancer.

## Introduction

Gastric Cancer (GC), the third highest mortality rate among malignant tumors and the fifth most common cancer, seriously threatens human health and life.^1^ Although numerous treatment methods such as endoscopic treatment, surgery, lymphadenectomy, chemoradiotherapy, and immunotherapy have been used to treat GC,^2^, ^3^, ^4^ the five-year survival rate in patients with early GC is more than 90 % while the five-year survival rate for patients with advanced GC is still low,^5^ and effective strategies for improving therapeutic effect and prognosis of patients with GC are urgently needed. The main cause leading to poor therapeutic effect in GC is the complicated molecular mechanisms of GC. Interestingly, some studies have revealed the association of Cystatin SN (CST1) a cysteine protease inhibitor that protects against allergen and viral, and AKT pathway an important pathway that controls many cellular processes such as survival, cell division, and autophagy in suppression and progression of GC, and these previous results provided great potential for GC targeted therapy.^6^^,^^7^ However, the detailed mechanism of CST1, and the AKT pathway in GC is still unclear, and it is urgent to conduct a related study to elucidate the role of CST1, and the AKT pathway in GC.

AKT pathway, an important signaling pathway regulating normal cellular processes, which is associated with many human cancers through modulating chemoresistance, apoptosis, autophagy, and metastasis.^8^ Given that the AKT pathway plays a crucial role in normal cellular processes and its aberrant inhibition or downregulation is closely involved in the suppression and progression of GC.^9^ Therefore, targeting and inhibiting the AKT pathway can significantly prevent the occurrence and progression of GC.

CST1, one of Type 2 (T2) cystatin protein superfamily,^10^ participates in numerous disease development by modulating the cellular processes including cell proliferation, migration, and invasion.^11^^,^^12^ Aberrant activation of the expression of CST1 can contribute to the development of GC by promoting tumor cell migration.^12^^,^^13^ Elevated CST1 is closely related to the progression of numerous diseases including airway eosinophilic inflammation in Asthma and breast cancer through modulating the AKT pathway.^14^^,^^15^ Both CST1 and AKT pathways are closely associated with the occurrence and progression of GC, but the role of the mechanism of CST1 controlling the AKT pathway in GC remains elusive.

Here, the authors established an in vitro model of GC to investigate the molecular mechanisms of CST1 promoting GC development through activating AKT pathway.

## Material and method

### Cell culture and treatment

The human gastric cancer cell line MGC-803 and normal gastric tissue cells were obtained from PromoCell Co., Ltd and cultured in DMEM medium (Thermo Fisher, USA) accompanied with the addition of 1 % penicillin/streptomycin and 10 % FBS (Biologic Industries), and the temperature was set at 37 °C and humidified air with 5 % CO_2_. Then, the obtained cell lines were divided into following four groups: control group (normal gastric tissue cells), model group (GC cell line), and CST1-activator group of which MGC-803 cells were induced by overexpression vector of CST1 (oe-CST1), and CST1-inhibitor group of which MGC-803 cells were treated by short hairpin RNA targeting CST1 (sh-CST1). All protocols used for this cell study adhere to the guidelines for the use of cell lines in biomedical research, and ethical approval (no. 2022092) for this study was obtained from the Institutional Review Board of the First Affiliated Hospital of Soochow University.

### Transwell assay

A transwell system featuring 0.4 μm chambers was employed to examine the invasive capabilities of trophoblasts. The obtained cells were suspended in a serum-free medium at a concentration of 5 × 10^4^ cells/mL. A 200 μL aliquot of the cell suspension was added to the upper chamber pre-coated with a Corning® Matrigel® Matrix diluted at a 1:8 ratio with RPMI-1640. Subsequently, 600 μL of complete medium containing 5 % FBS was introduced into the lower chamber. Following a 24 h incubation period at 37 °C, cells in the upper chambers were meticulously eradicated. Cells that invaded through the membrane were treated with 4 % paraformaldehyde fixation and stained using 0.1 % crystal violet. The invaded cells were quantified by examining five random fields on the lower membrane surface at ×200 magnification using an inverted microscope. Images of the invaded cells were captured with a Leica microscope and subsequently evaluated.

### Scratch assay

Suck off the original culture medium and clean the obtained cells with PBS. Add a volume of 1 mL pancreatic enzyme digestion solution to digest cells. Collect cell suspension in a centrifuge tube, and centrifuge at room temperature for 5 min at 800‒1000 rpm/min. Inoculate approximately at a density of 5 × 10^5^/well onto a culture plate, then make a scratch on the plate using a 20 μL tip. Observed the width of the trace under a microscope and took photos at 0 and 24 h, then imported them into the software for analysis.

### Western blotting

The obtained protein from normal gastric tissue and GC cells was subjected to 10 % SDS-PAGE and loaded, transferred to PVDF membrane, and washed with TBST for 5 min. Primary antibody (1:2000; Bioworld Techmology, Inc., China) and GAPDH (1:1000; Bioworld Techmology, Inc., China) were added and incubated overnight at 4 °C. After washing with TBST 3 times (10 min/time), a secondary antibody (1:10,000; Bioworld Techmology, Inc., China) was added and blocked for 2 h at room temperature. After washing with TBST 3 times (10 min/time), ECL luminescent reagent was added for development, and the gray value of the bands was analyzed.

### qRT-PCR

Total RNA was extracted utilizing the TRIzol Reagent (Beyotime, Shanghai) according to the manufacturer's guidelines. mRNA and cDNA were produced using the mRNA Reverse-Transcription Kit (Beyotime, Shanghai). The quantitative assays were performed using the SYBR Green PCR Mix (Vazyme Biotech, Shanghai) with a Real-Time PCR System. To calculate the relative mRNA expression levels, the 2^−ΔΔCt^ method was employed to determine the relative expression values, which were normalized to GAPDH. The test was done three times. The sequence of the CST1 primer was (Forward: 5′-CGGGTGGCATCTATAACGCA-3′ and reverse: 5′-GTCTGTTGCCTGGCTCTTAGT-3′), and the AKT was (Forward: 5′- AGCGACGTGGCTATTGTGAAG-3′ and reverse: 5′-GTACTGGTCTGGATAG-3′), and the GAPDH was (Forward: 5′-CATGTGGGCCATGAGGTCCACCAC-3′ and reverse: 5′-GGGAAGCTCACTGGCATGGCCTTCC-3′).

### Flow cytometry assay

According to the manufacturer's direction, the obtained cells were analyzed using flow cytometry. Briefly, the cells were collected and stained with Annexin V-FITC and Propidium Iodide (PI) in the darkness, and then Apoptosis levels in all groups were analyzed using a flow cytometer.

### Statistical analysis

Prism 8 was used to analyze the results. All data were presented as mean ± SD and repeated 3 times at least. To assess the significance of disparities between the two groups, a *t*-test was used, while a one-way ANOVA was employed for comparing three or more groups, and a *p*-value <0.05 was accepted as statistically significance.

## Results

### Comparison of the invasive capabilities

**Fig. 1:**
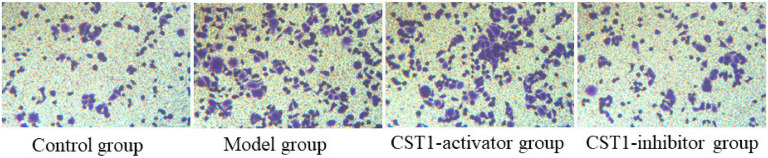
Invasive capacity for normal gastric tissue cells and GC cells were detected using transwell assay.

To evaluate the invasive capabilities of normal gastric tissue cells and GC cell line induced by oe-CST1 or sh-CST1, a transwell assay was performed, shown in Fig. 1. These results show that invasive capacities in the model group and CST1-activator group was significantly increased compared to that in the control group, while knockdown of CST1 in the CST1-inhibitor group clearly decreased the invasive capacities compared to that in the model group, and the *p*-value is Statistical different (*p* < 0.05).

### Comparison of the metastatic capabilities

**Fig. 2:**
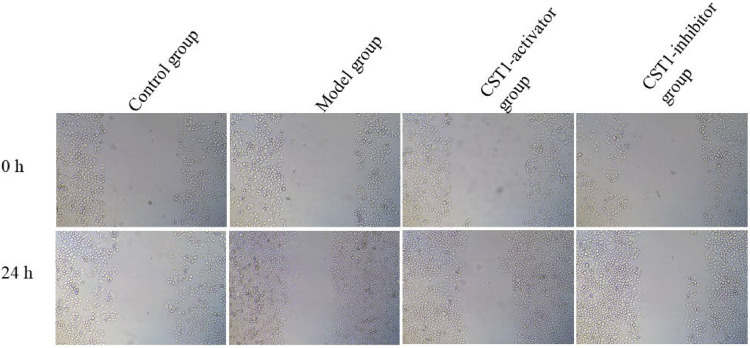
The metastatic capabilities for normal gastric tissue cells and GC cells were detected using scratch assay.

To assess the metastatic capabilities of normal gastric tissue cells and GC cell line-induced by oe-CST1 or sh-CST1, scratch assay was performed, depicted in Fig. 2. These results show that the metastatic capabilities at 0 h among the control group, model group, CST1-activator group, and CST1-inhibitor group are similar. However, the metastatic capabilities at 24 h in the model and CST1-activator group was obviously increased compared to that in the control group, while the metastatic capabilities in the CST1-inhibitor group were clearly decreased compared to that in the model group and the *p*-value are Statistical differences (*p* < 0.05).

### Effect of CST1 and AKT protein in gastric cancer

**Fig. 3:**
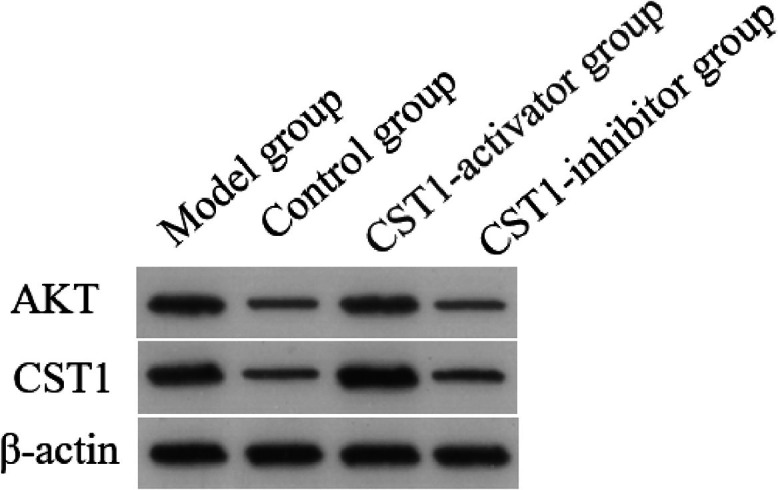
The expression level of CST1 and AKT protein in normal gastric tissue and GC cells was determined using Western-blotting.

Western-blotting was utilized to assess the expression level of CST1 and AKT protein in normal gastric tissue and GC cells, as shown in Fig. 3. The findings indicate that both CST1 and AKT protein levels were markedly elevated in the model and CST1-activator groups compared to the control group; however, the levels of these proteins were significantly reduced in the CST1-inbihitor group when contrasted with the model group, and the *p*-value is Statistical differences (*p* < 0.05). These results indicated upregulation of CST1 and AKT protein was correlated with the occurrence and progression of GC.

### Effect of CST1 and AKT mRNA in gastric cancer

**Fig. 4:**
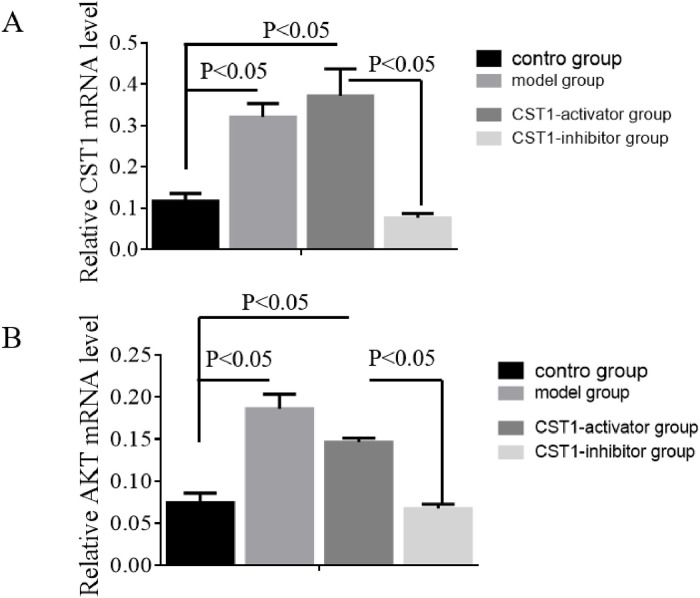
The expression level of CST1 and AKT mRNA in normal gastric tissue and GC cells was determined using qRT-PCR.

qRT-PCR was utilized to assess the expression level of CST1 and AKT mRNA in normal gastric tissue and GC cells, depicted in Fig. 4. These results show that the expression level of CST1 and AKT mRNA in the model and CST1-activator group was obviously higher than that in the control group. However, the expression level of CST1 and AKT mRNA was significantly decreased in the CST1-inhibitor group compared to that in the model group, and the *p*-value are Statistical differences (*p* < 0.05). These results indicated upregulation of CST1 and AKT mRNA was correlated with the occurrence and progression of GC.

### Apoptosis level in gastric cancer cells

**Fig. 5:**
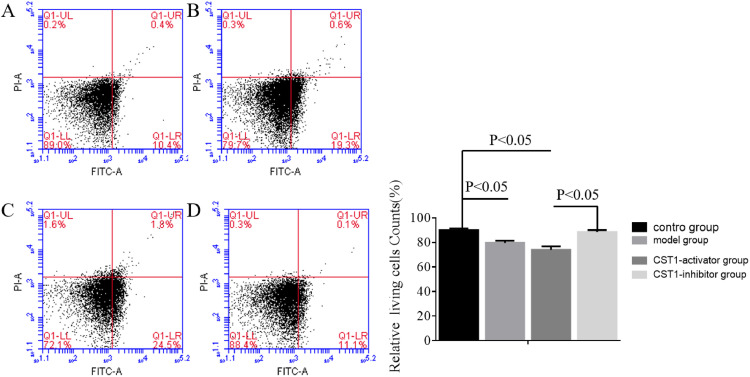
Apoptosis levels in normal gastric tissue and GC cells were detected using Flow Cytometry (FCM). (A‒D) Presented apoptosis level of control group, model group, CST1-activator group, and CST1-inhibitor group, respectively.

Flow Cytometry (FCM) was utilized to measure the apoptosis level in normal gastric tissue and GC cells, as shown in Fig. 5. These results show that relative living cell counts in the model and CST1-inhibitor group were less than that in control group (*p* < 0.05). However, relative living cells counts were increased in the CST1-inhibitor group compared to that in the model group, and the *p*-value are Statistical differences (*p* < 0.05). These results indicated upregulation of CST1 and AKT contributed to relative living cell counts in normal gastric tissue and GC cells.

## Discussion

The high mortality rate among individuals with gastric cancer is primarily due to the aggressive spread and metastasis of the cancer cells, which is the predominant cause of fatality in these patients.^16^ Currently, numerous therapeutic methods have been used to treat gastric cancer, but gastric cancer cannot be cured and only related symptoms are prevented, and complex mechanism of gastric cancer contributed to poor therapeutic effect.^5^ CST1 was well-known as a cysteine protease inhibitor that protects against allergen and viral, and was associated with the gastric cancer process. More and more studies^17^^,^^18^ have indicated that AKT pathways are involved in the development of gastric cancer, their role and mechanism in gastric cancer, yet, were still elusive. More trials should be conducted to clarify the mechanism of gastric cancer. The results in this study mainly investigate the molecular mechanisms of CST1 promoted gastric cancer development by activating AKT pathway. These outcomes suggested that the activation of AKT pathway can promote the gastric cancer process, and CST1 promoted gastric cancer development via AKT pathway.

AKT pathway, a crucial pathway controlling numerous cellular processes, was associated with cancer occurrence and progression by controlling the downstream targets.^19^^,^^20^ Although many studies have investigated the effect of AKT pathway in gastric cancer, and the results in present study indicated that activation of AKT pathway could promote the development of gastric cancer. The present results suggested that activation of AKT pathway can promote the development of gastric cancer, while inhabitation of AKT pathway can alleviate the gastric cancer progress. A trial by Rong L^9^ has investigated the role and mechanism of AKT pathway expression in gastric cancer, concluding that inhibition of AKT pathway alleviates gastric cancer growth, and these results are consistent with those described in this study. These results indicated inhibition or activation of the AKT pathway was correlated with gastric cancer progress.

CST1 is well-known as a cysteine protease inhibitor primarily participating in the development of cancer, and numerous studies have demonstrated that upregulation of CST1 promotes cancer development. Currently, the detailed mechanism of CST1 in gastric cancer is unclear. These results have investigated the role of CST1 in gastric cancer and these outcomes showed that down-regulation of CST1 alleviated gastric cancer, while upregulation of CST1 expression can promote the development of gastric cancer. Numerous studies^9^^,^^13^^,^^21^ have reported upregulation of CST1 could contribute to gastric cancer by accelerating cancer cell metastasis. In addition, the results suggested inhibition of CST1 prevented the development of gastric cancer development via the AKT pathway, and few studies elucidated the association of CST1 regulating the AKT pathway in gastric cancer. The results indicated that CST1 regulating the AKT pathway was correlated with gastric cancer progress.

A limitation of the present study was that the authors only conducted an in vitro study to investigate the molecular mechanisms of CST1 regulating AKT pathway in gastric cancer, and further clinical study was needed to manage. Recent research has established a connection between CST1′s influence on the AKT signaling pathway and the advancement of gastric cancer, indicating that CST1 can promote the gastric cancer process by targeting the AKT pathway.

## Ethics approval and consent to participate

All animal and cell experiments were conducted in compliance with the ARRIVE guidelines and in accordance with the National Institutes of Health Guide for the Care and Use of Laboratory Animals. The ethics approval was reviewed and approved by The Punnan Branch of Renji Hospital, Shanghai Jiaotong University School of Medicine (no. 2022092).

## Consent for publish

All of the authors have consented to publish this research.

## Funding

The mechanism research of lncRNA-C17orf97 promoted gastric cancer development of regulating the AKT3 pathwayIncRNA-C17orf97 (No. PN2021A5).

## CRediT authorship contribution statement

**Lei Zhang:** Conceptualization, Methodology, Software, Validation, Formal analysis, Investigation, Resources, Data curation, Writing – original draft, Writing – review & editing, Visualization, Supervision, Project administration. **Dongmei Wang:** Methodology, Software, Validation, Formal analysis, Investigation, Resources, Data curation, Writing – review & editing, Visualization, Supervision, Project administration. **Liqun Zhang:** Conceptualization, Methodology, Software, Validation, Formal analysis, Investigation, Resources, Data curation, Writing – original draft, Writing – review & editing, Supervision. **Lini Zhu:** Conceptualization, Methodology, Software, Validation, Formal analysis, Resources, Writing – review & editing, Visualization, Supervision, Project administration, Funding acquisition.

## Conflicts of interest

The authors declare no conflicts of interest.
